# Hybrid Deflection of Spoiler Influencing Radar Cross-Section of Tailless Fighter

**DOI:** 10.3390/s21248459

**Published:** 2021-12-18

**Authors:** Zeyang Zhou, Jun Huang

**Affiliations:** School of Aeronautic Science and Engineering, Beihang University, Beijing 100191, China; junh@china.com

**Keywords:** electromagnetic scattering, radar stealth, tailless aircraft, spoiler control surface

## Abstract

With the continuous development of advanced fighters towards tailless and flying wing layouts, diverse control surfaces have become the mainstream design. To study the influence of spoiler control surface on the radar cross-section (RCS) of a tailless fighter, a calculation method is presented. The deflection angle of the spoiler is controlled by the fixed mode, linear mode, and smooth mode. The results show that the opening action of the spoiler will break the original stealth characteristics of the aircraft at the key azimuth angles of the head and tail. As the elevation angle increases, this adverse effect will spread to the side. The influence of the different dynamic deflection modes of the spoiler on the aircraft RCS is analyzed. Compared with the linear dynamic deflection mode, the smooth dynamic deflection mode is conducive to the reduction in the average RCS at the given head azimuth. The presented method is effective to study the influence of the spoiler deflection on the electromagnetic scattering characteristics of the tailless aircraft.

## 1. Introduction

The design of a new generation of fighter jets has more and more stringent requirements for stealth. In order to make its appearance more concise and complete, measures such as tailless layout, spoilers, split ailerons, and flaps are favored [1,2,3]. The influence of the aerodynamic characteristics of the opening and closing of the spoiler has always been the focus of attention, and the process of its rotation around the axis will bring dynamic electromagnetic scattering characteristics to the aircraft. Studying the influence of the spoiler’s action on the aero/stealth comprehensive characteristics of the advanced fighter has important engineering value.

Spoiler slot deflector is an important control surface of innovative control effector (ICE) aircraft, and its opening action can affect the yaw moment and aerodynamic drag of the aircraft [4]. When flying at a low angle of attack, the non-linear change of the pitching moment of this type of tailless aircraft may become unstable [5,6]. The computational fluid dynamics (CFD) method based on Reynolds-averaged Navier–Stokes (RANS) was used to study the influence of the upward deflection of the spoiler [7]. Menter k-ω shear stress transport turbulence model was adopted to deal with the changes in the control characteristics of the flying wing by the split rudder and spoiler [8,9]. For multi-control surface tailless flying wing aircraft [10,11], asymmetric deflection of the split drag rudder can greatly increase the pitch and roll moments [12]. The control surface may form a certain gap with the stabilizer during the manufacturing process [13,14], which will affect the aerodynamic force, torque, and control efficiency of the aircraft [15]. The fully turbulent and transitional RANS simulates the pitch characteristics of a flying wing with a low RCS at a low angle of attack [16]. Differential leading edge flaps, spoilers, split rudders, full-moving wingtips, and air brakes are all promising control devices for ICE aircraft [17,18,19]. Both ailerons and rudder can provide sufficient control over the lateral stability of the hypersonic waverider, but the aircraft may benefit from the increased rudder size [20]. These control surfaces have a substantial impact on the aerodynamic characteristics, stability, and control efficiency of the aircraft. One thing to note is that they will change the original shape of the aircraft when they are turned on, which may produce a large electromagnetic scattering effect in some directions.

With the increase in comprehensive research on aerodynamics and stealth performance, the electromagnetic scattering characteristics of flying wings with different configurations have also received more and more attention [21,22,23]. Air dominance UAV is an important development direction for manned/unmanned fighter jets in the future. A small aspect ratio duck-type flying wing aerodynamic layout is therefore presented [24]. The influence of actions including canards and spoilers on the dynamic RCS of the aircraft is yet to be resolved [25,26]. Considering that the modern aircraft is accompanied by complex flow field interference during the high angle of attack post-stall maneuvering, diversified design of control surface and intelligent flight control also face more challenges [27]. Different spoiler configurations and the continuous opening of the control surface will affect the airflow at the tail of the aircraft, which will affect the mixing effect of the hot and cold airflow near the nozzle [28,29]. Through the control surface and thrust vector, the fighter can be quickly pulled up [30], and this series of actions and attitude changes will also bring about major changes to the aircraft’s radar/infrared stealth characteristics [31,32]. The research and development of a new generation of fighter jets has led the innovation and development of various technologies in the field of aircraft design [33], including aerodynamics, stability, stealth characteristics [34,35], and control theory.

It is evident that a lot of research work has been carried out in the literature on the influence of spoiler and other control surfaces on the aerodynamic characteristics of aircraft. For advanced fighters with flying wings or tailless layouts, the focus is on stability and control laws. Moreover, some experimental studies did not consider the cabin of the control surface when turning on the spoiler or the air brake, even though this cabin was relatively shallow. In fact, the entire process of opening and closing the spoiler is constantly changing the shape of the aircraft, which also changes the deflection of incident radar waves in a fixed direction; thus, this control surface will also bring dynamic RCS to the aircraft. In order to achieve high-density RCS time results, it takes a lot of time and many steps for the conventional methods or algorithms to complete the repeated process of modeling and grid. The innovation of this paper is to efficiently solve the dynamic RCS characteristics of the tailless fighter in the hybrid deflection modes of the spoiler. This is undoubtedly of academic significance and engineering value for the actual survivability evaluation of advanced tailless fighters.

In this paper, the calculation method is presented in Section 2. Models are built in Section 3. RCS results are provided and discussed in detail in Section 4. Finally, the full text is summarized in Section 5.

## 2. Calculation Method

**Figure 1:**
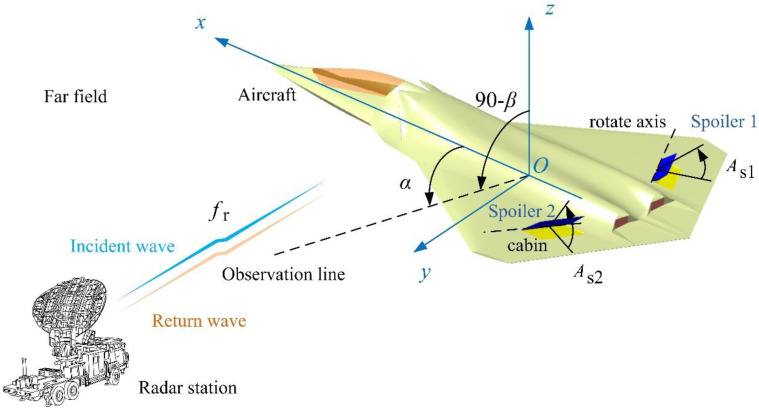
Schematic of influence of spoiler deflection on aircraft electromagnetic scattering characteristics, drawn using Microsoft Visio 2007.

The schematic diagram of the influence of spoiler deflection on the electromagnetic scattering characteristics of the aircraft is shown in Figure 1, where *α* is the azimuth angle between the radar station and the aircraft, *β* is the elevation angle between the radar station and the aircraft, *A*_s1_ is the deflection angle of spoiler 1, *A*_s2_ is the deflection angle of spoiler 2, and *f*_r_ is the radar wave frequency. The calculation method includes two modules: hybrid deflection control and instantaneous RCS calculation.

### 2.1. Hybrid Deflection Control

The deflection modes of spoiler control surface usually include unilateral deflection, bilateral synchronous deflection, constant opening mode with fixed angle, and dynamic continuous deflection in a short time. Therefore, when the spoiler is in dynamic deflection, the deflection angle of the spoiler can be defined as follows:

(1) $$A_{s}=\left\{\begin{array}{l} \omega_{s}\cdot t\\ A_{k}\sin\left(\omega_{k}\cdot t-\frac{\pi }{2}\right)+C_{k} \end{array}\right.\begin{array}{cc} & \begin{array}{l} \begin{array}{cc} \mathrm{mode} & M1 \end{array}\\ \begin{array}{cc} \mathrm{mode} & M2 \end{array} \end{array} \end{array}$$

where *t* is time, M1 indicates linear deflection mode, M2 indicates smooth deflection mode, *ω*_s_ is the angular velocity of deflection, and *A*_k_, *ω*_s_, and *C*_k_ are the control coefficients of M2 mode. In M1 mode, the deflection angle of the spoiler changes linearly, while in smooth mode, the spoiler deflects according to a sinusoidal-like curve.

When the spoiler is at a fixed deflection angle:

(2) $$A_{s}=A_{s0}\begin{array}{cc} , & A_{s0}\in \left[\begin{array}{cc} 0, & 90 \end{array}\right]\deg \end{array}$$

where *A*_s0_ is the value given in the domain, then this mode in which the deflection angle remains constant is called the fixed mode and is recorded as M0 mode. In M0 mode, the deflection modes of the spoiler include unilateral deflection and bilateral deflection.

When spoiler 1 or 2 deflects, the scattering sources on the upper surface of the aircraft are constantly changing for the electromagnetic waves in the fixed incident direction. For spoiler 1, its rotation axis is transformed to coincide with the *y* axis of the *Oxyz* coordinate system. First, select a point *P*_s1_ on the rotation axis of spoiler 1:

(3) $$M^{z}\left(m_{s1}\left(t=0\right)\right)=M\left(z\left(m_{s1}\left(t=0\right)\right)-z\left(P_{s1}\right)\right)$$

(4) $$M^{yz}\left(m_{s1}\left(t=0\right)\right)=M^{z}\left(y\left(m_{s1}\left(t=0\right)\right)-y\left(P_{s1}\right)\right)$$

(5) $$M^{xyz}\left(m_{s1}\left(t=0\right)\right)=M^{yz}\left(x\left(m_{s1}\left(t=0\right)\right)-x\left(P_{s1}\right)\right)$$

where *m*_s1_ represents the model of spoiler 1, *t* is time, ***M*** represents the grid matrix of the model, and superscripts indicate different transform operations. The selection of reference points here may not be unique, and the purpose is to move the spoiler to the origin. Generally, the point near the origin on the rotation axis of the spoiler is selected. Rotate the obtained spoiler model around the *z* axis:

(6) $$M^{r,z}\left(m_{s1}\left(t=0\right)\right)=\left[\begin{array}{ccc} \cos A_{z1} & -\sin A_{z1} & 0\\ \sin A_{z1} & \cos A_{z1} & 0\\ 0 & 0 & 1 \end{array}\right]\cdot M^{xyz}\left(m_{s1}\left(t=0\right)\right)$$

where *A*_z1_ is the angle of rotation about the *z* axis. Due to the integrated design of the spoiler shape and the wing, the rotation operation is required. Rotate the obtained spoiler model around the *x* axis:

(7) $$M^{r,x}\left(m_{s1}\left(t=0\right)\right)=\left[\begin{array}{ccc} 1 & 0 & 0\\ 0 & \cos A_{x1} & -\sin A_{x1}\\ 0 & \sin A_{x1} & \cos A_{x1} \end{array}\right]\cdot M^{r,z}\left(m_{s1}\left(t=0\right)\right)$$

where *A*_x1_ is the angle of rotation about the *x* axis.

When this spoiler opens upwards, its dynamic model can be expressed as:

(8) $$M^{r,y}\left(m_{s1}\left(t\right)\right)=\left[\begin{array}{ccc} \cos\left(-A_{s1}\left(t\right)\right) & 0 & -\sin\left(-A_{s1}\left(t\right)\right)\\ 0 & 1 & 0\\ \sin\left(-A_{s1}\left(t\right)\right) & 0 & \cos\left(-A_{s1}\left(t\right)\right) \end{array}\right]\cdot M^{r,x}\left(m_{s1}\left(t=0\right)\right)$$

For spoiler 2, the transformation operations are similar to those of spoiler 1. For details, please refer to Equations (A1)–(A6) in Appendix A. By combining the spoiler dynamic model with the fuselage model, the dynamic model of the aircraft can be obtained as follows:

(9) $$M\left(m_{\mathrm{airc}}\left(t\right)\right)=\left[M\left(m_{b}\left(t\right)\right);M\left(m_{s1}\left(t\right)\right);M\left(m_{s2}\left(t\right)\right)\right]$$

where *m*_b_ is the model of aircraft fuselage, and *m*_airc_ is the aircraft model. When the attitude angle of the aircraft does not change:

(10) $$M\left(m_{\mathrm{airc}}\left(t\right)\right)=\left[M\left(m_{b}\left(t=0\right)\right);M\left(m_{s1}\left(t\right)\right);M\left(m_{s2}\left(t\right)\right)\right]$$

At this time, the fuselage can be regarded as always fixed in the current reference system.

### 2.2. Instantaneous RCS Calculation

This aircraft model has a large area of curved surfaces and multiple edges, so PO (physical optics) and PTD (physical theory of diffraction) are used to calculate its instantaneous RCS. Then, the dynamic RCS response of the aircraft can be calculated as:

(11) $$\sigma \left(t\right)={\left|\sum_{i=1}^{N_{f}}\left(\sqrt{\sigma_{F}\left(t\right)}\right)\begin{array}{c} i \end{array}+\sum_{j=1}^{N_{e}}\left(\sqrt{\sigma_{E}\left(t\right)}\right)\begin{array}{c} j \end{array}\right|}^{2}\begin{array}{c} S_{I}\left(t\right) \end{array}$$

where *N*_f_ is the number of facets, *N*_e_ is the number of edges, and *S*_I_(*t*) is the illuminated surface extracted from the dynamic grid matrix. *σ* is radar cross-section, subscript F denotes facet contribution, and subscript E denotes edge contribution. For more information about PO and PTD, please refer to the literature [22,25].

In order to visually analyze and judge the scattering effect of the spoiler or aircraft, a custom surface scattering intensity could be written as follows:

(12) $$I_{\mathrm{ss}}\left(i\right)=K_{\mathrm{cd}}\left(B_{cm}-B_{cn}\right)\frac{\sigma_{f}\left(i\right)-\sigma_{\mathrm{fn}}}{\sigma_{\mathrm{fm}}-\sigma_{\mathrm{fn}}}+K_{\mathrm{cd}}\cdot B_{cn}$$

where *I*_ss_(*i*) is custom surface scattering intensity, *σ*_f_ is the facet RCS under the current condition, the *i* in brackets is the facet number, *σ*_fn_ is the minimum of the facet RCS, and *σ*_fm_ is the maximum of the facet RCS. *K*_cd_ is the color depth control factor, *K*_cd_ ∈ [0.1, 10], where the smaller the KCD, the easier it is to capture the strong scattering source position of the surface. *B*_cm_ and *B*_cn_ represent the upper and lower boundaries of the current color window, respectively.

### 2.3. Method Validation

**Figure 2:**
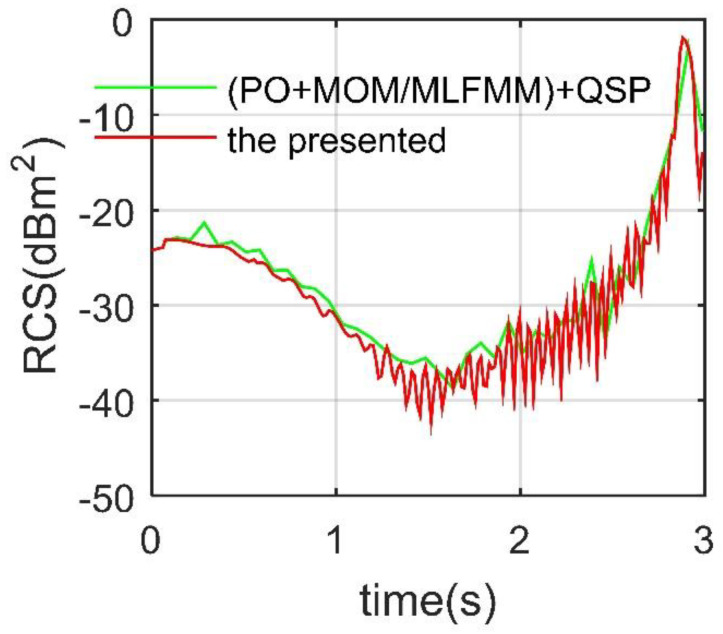
Verification of the presented calculation method for spoiler 1, *α* = 10°, *β* = 0°, *f*_r_ = 10 GHz, HH, drawn using Microsoft Visio 2007 and MATLAB 2015A.

The presented method used to calculate the dynamic RCS is verified as shown in Figure 2, where *ω*_s1_ is set to 0.5236 rad/s, and QSP is the quasi-static principle, which decomposes the deflection of the spoiler into a series of discrete states. PO + MOM (method of moment)/MLFMM (multilayer fast multipole method) in FEKO is used to calculate the RCS of the target [36,37]. HH means radar wave horizontal polarization. It can be seen that the changes in the dynamic electromagnetic scattering characteristics of the spoiler reflected by the two RCS curves are generally similar, while the RCS given by the presented method is more consistent in time and has clearer fluctuation details. At the same time, the RCS change of the deflection of a single spoiler at an azimuth angle of 10° is obviously huge, with a maximum difference of 40.1986 dBm^2^. Compared with the conventional methods, the calculation method presented in this paper shows the details of dynamic RCS curve more continuously and efficiently. This figure represents the verification of RCS time calculation results by the presented calculation method under azimuth. Note that the verification of RCS azimuth results is shown in Figure A4 of Appendix A. These results indicate that it is feasible for the presented method to analyze the dynamic electromagnetic scattering characteristics of the spoiler, and it is necessary to study the influence of the spoiler deflection on the stealth characteristics of the aircraft.

## 3. Models of Spoiler and Aircraft

**Figure 3:**
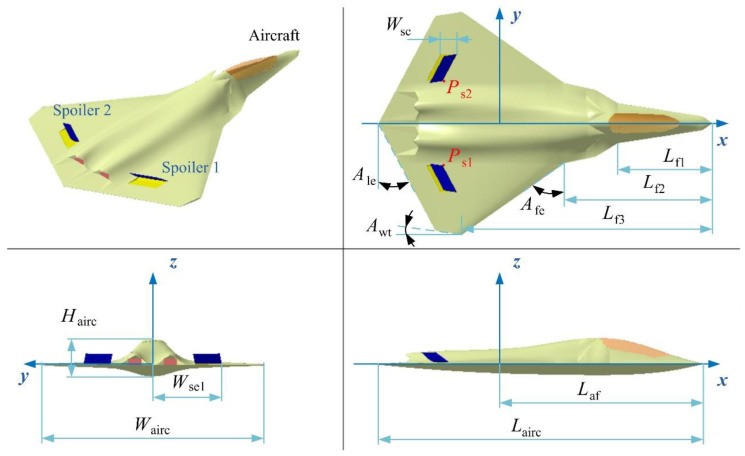
Model establishment of aircraft and its spoilers, *A*_s1_ = *A*_s2_ = 45°, drawn using Microsoft Visio 2007 and CATIA V5R20.

The model of the aircraft is built as shown in Figure 3, where two spoilers are set on the upper surface of the wing and are symmetrical about the *xz* plane. The leading and trailing edges of the spoilers are parallel to the trailing edge of the wing. *L*_airc_, *W*_airc_, and *H*_airc_ are the length, width, and height of the aircraft, respectively. *A*_fe_, *A*_le_, and *A*_wt_ are the sweep angle of the leading edge of the wing, the forward sweep angle of the trailing edge of the wing, and the wing tip cut angle, respectively. *L*_af_, *L*_f1_, *L*_f2_, and *L*_f3_ indicate the length of the main section of the fuselage. *W*_sc_ represents the width of the spoiler cabin, and *W*_se1_ is the distance from the outer end of the spoiler 1 cabin to the plane of symmetry of the aircraft.

The main dimensions of the aircraft are given as shown in Table 1, including the length of each part of the fuselage, wing chamfer, and fuselage width, etc. In addition, the coordinates of point *P*_s1_ in *Oxyz* are (−3.6, −1.9, 0.05188) m, where the deflection axis of the spoiler is parallel to its leading edge.

**Figure 4:**
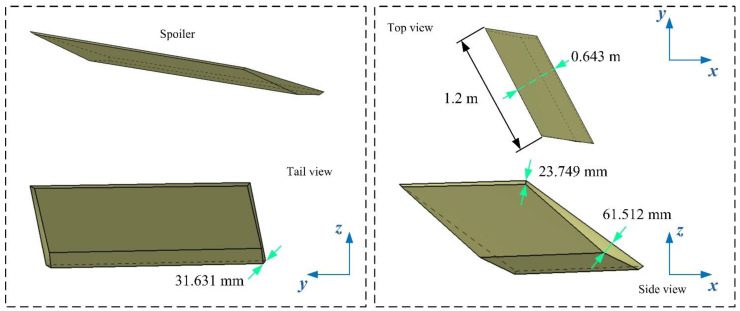
Details of the spoiler 1 model, *A*_s1_ = 45°, drawn using CATIA V5R20.

The details of the spoiler model are presented in Figure 4, where the leading and trailing edges of the spoiler are parallel, and both are parallel to the trailing edge line of the wing. Due to the flat and sharp shape of the entire spoiler, the edge lines are deepened to black to distinguish the front from the back. The configuration of the whole spoiler adopts the low scattering feature design, and thus the narrow surface of the edge is not perpendicular to the upper surface of the spoiler.

**Figure 5:**
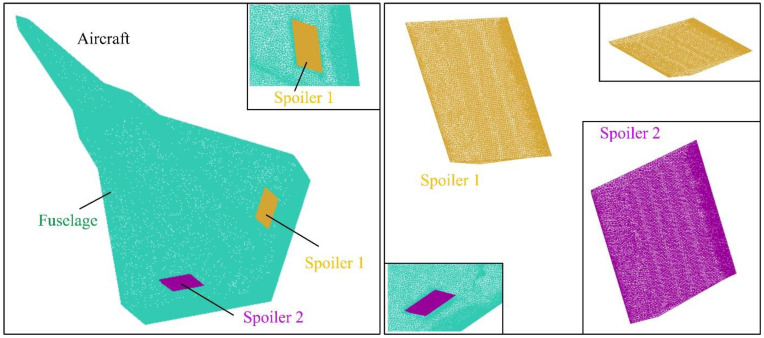
Mesh of aircraft fuselage and spoiler, *A*_s1_ = *A*_s2_ = 45°, drawn using Microsoft Visio 2007 and ICEM CFD 16.0.

High-precision unstructured grid technology is used to generate grids on the surface of fuselage and spoiler as shown in Figure 5, where the mesh density increasing technology for line/surface is adopted to improve the local mesh quality, including the front and rear edges of the wing, fuselage edge lines, air inlet and spoiler edges, etc. The grid size of each part of the aircraft is provided in Table 2, where the global minimum size is used to improve the mesh quality of some surface and edge details.

## 4. Results and Discussion

**Figure 6:**
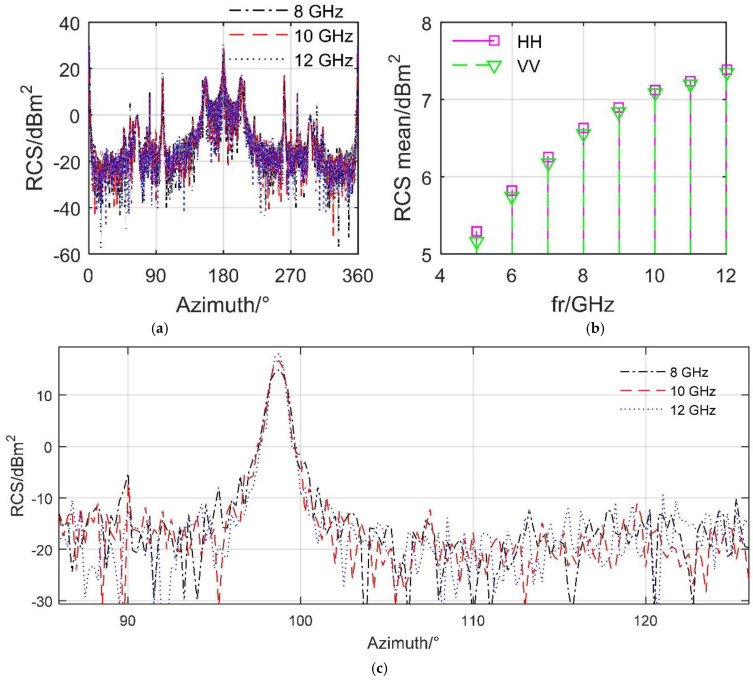
Aircraft radar cross section at different radar wave frequencies, *m*_0_, spoiler not opened, *β* = 0°, drawn using MATLAB 2015A. (**a**) RCS curve, HH. (**b**) RCS mean, (**c**) RCS curve (zoom in), HH.

**Figure A1:**
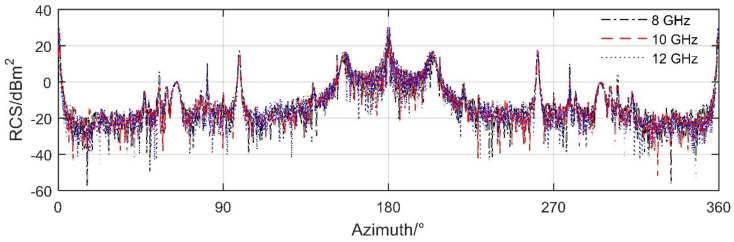
Aircraft RCS at different radar wave frequencies, *m*_0_, spoiler not opened, *β* = 0°, drawn using MATLAB 2015A.

Figure 6 presents that as the frequency of the incident radar wave increases, the aircraft’s RCS level gradually increases, including peak and average values, where VV means vertical polarization. Here, *m*_0_ is a closed model of the aircraft, and the spoiler is not opened at this time. At an azimuth angle of 98.75°, the aircraft’s RCS curve has a small peak, where the peak value of the 8 GHz curve is 14.95 dBm^2^, that of the 10 GHz curve is 16.66 dBm^2^, and that of the 12 GHz curve is 18.09 dBm^2^. Refer to Figure A1 in Appendix A for more detailed RCS data. For the results of the average RCS, it can be seen that as the radar wave frequency increases from 5 GHz to 12 GHz, the RCS mean of the aircraft gradually increases while the speed of the mean increase decreases gradually. Noting that, when making statistics on the mean RCS value of a curve, first convert the RCS (dBm^2^) of each point into RCS (m^2^), then calculate the mean value of these RCS (m^2^), and then convert this mean value into RCS (dBm^2^). When *f*_r_ = 5 GHz, the average RCS under HH is 5.286 dBm^2^, that under VV is 5.166 dBm^2^, and when *f*_r_ increases to 12 GHz, the RCS mean exceeds 7.349 dBm^2^. These results show that there are obvious differences in the electromagnetic scattering characteristics of aircraft under different radar wave frequencies. In order to compare the dynamic influence brought by the spoiler under the same incident wave conditions, the following calculations about RCS are all carried out at 10 GHz, and the radar wave is horizontally polarized.

### 4.1. Unilateral Fixed Deflection

**Figure 7:**
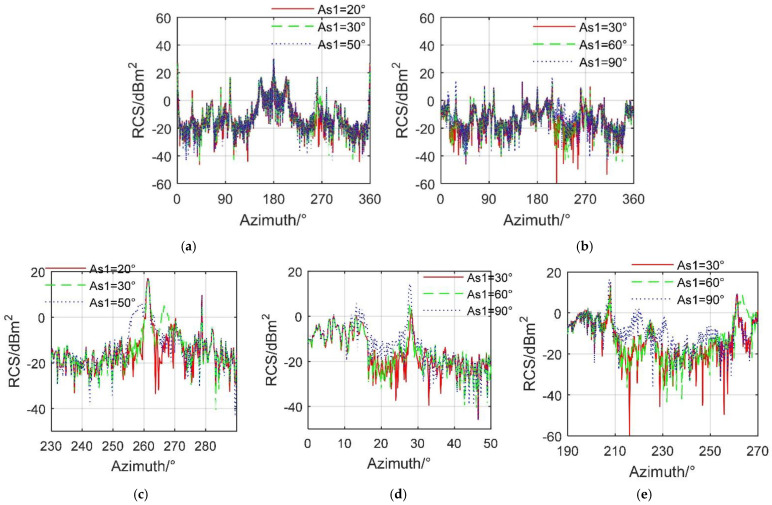
RCS of the aircraft with fixed deflection of spoiler 1, *A*_s2_ = 0°, HH, M0 mode, drawn using MATLAB 2015A. (**a**) RCS at *β* = 0°, (**b**) RCS at *β* = 10°, (**c**) RCS at *β* = 0° (zoom in), (**d**) RCS at *β* = 10°(zoom in), (**e**) RCS detail, *β* = 10°(zoom in).

Figure 7 shows that increasing the deflection angle of the one-sided spoiler can significantly change the radar cross section of the aircraft at certain azimuth angles. For *β* = 0° and *A*_s2_ = 0°, the three RCS curves are roughly similar, and the average value of RCS is also around 7.11 dBm^2^. In the azimuth range of 254°–259.8°, the RCS curve at *A*_s1_ = 50° is significantly higher than the other two, because the angle between the deflection axis of the spoiler and the radar wave is relatively large, and the angle between the normal direction of the back of the spoiler and the radar wave is small, which makes these areas easy to form strong scattering sources under the current azimuth angle. When *β* = 10° and *A*_s2_ = 0°, the electromagnetic scattering level of the aircraft is greatly reduced, and the influence of the spoiler on the aircraft RCS is enhanced. The mean value of the RCS curve when *A*_s1_ = 30° is −6.0705 dBm^2^, while those of *A*_s1_ = 60° and *A*_s1_ = 90° are −5.5965 dBm^2^ and −4.1129 dBm^2^, respectively. In the ranges of 11.75°–31° and 206.3°–252.25° azimuth angle, the RCS of the *A*_s1_ = 90° curve is obviously much larger than those of the other two. This is mainly due to the fact that the upright spoiler has a weaker ability to deflect incident waves, compared with the other two deflection angles. These results show that the influence of a single spoiler deflection on the electromagnetic scattering characteristics of the aircraft cannot be ignored.

### 4.2. Bilateral Fixed Deflection

**Figure 8:**
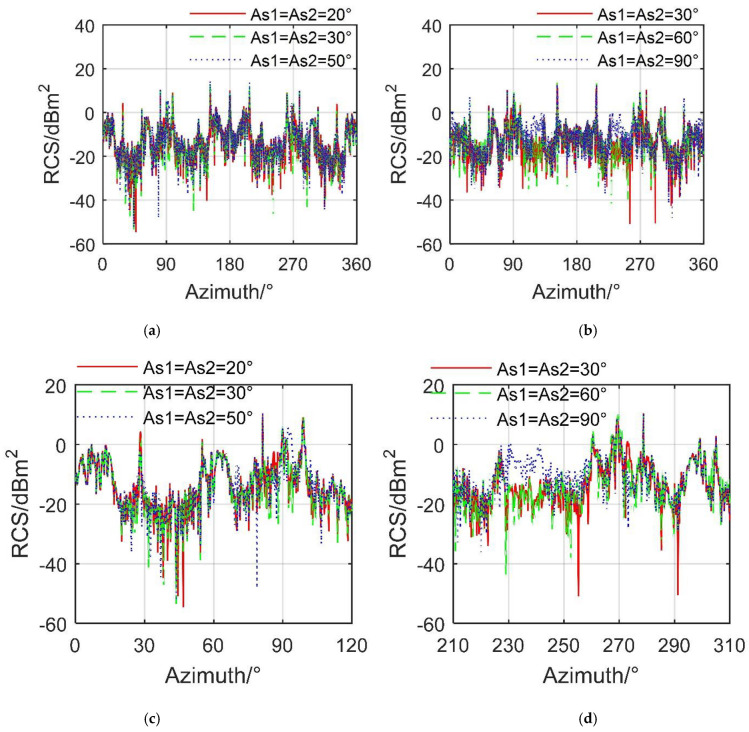
RCS of the aircraft with fixed deflection of bilateral spoiler, HH, M0 mode, drawn using MATLAB 2015A. (**a**) RCS at *β* = 10°, (**b**) RCS at *β* = 15°, (**c**) RCS at *β* = 10° (zoom in), (**d**) RCS at *β* = 15°(zoom in).

**Figure A2:**
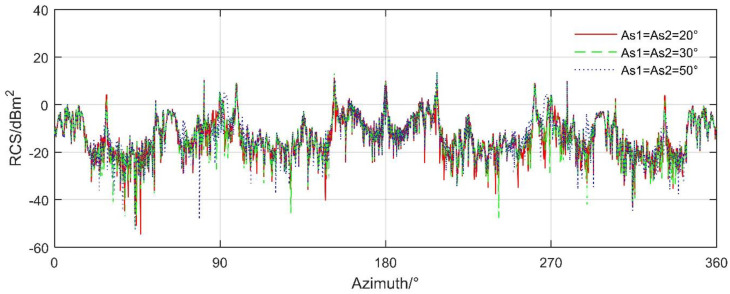
RCS of the aircraft, fixed deflection of spoiler 1, *β* = 10°, *A*_s2_ =0 °, HH, drawn using MATLAB 2015A.

**Figure A3:**
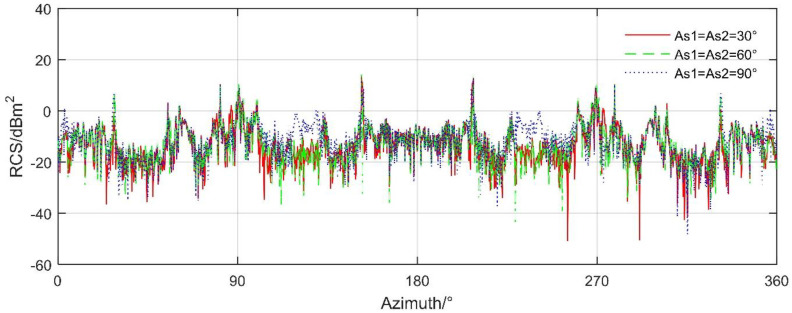
RCS of the aircraft, fixed deflection of spoiler 1, *β* = 15°, *A*_s2_ = 0°, HH, drawn using MATLAB 2015A.

**Figure A4:**
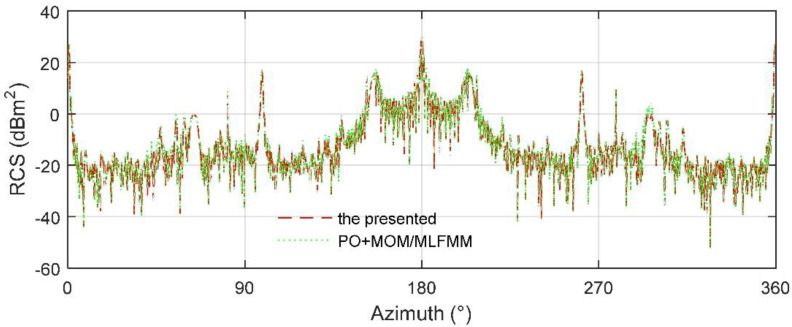
Verification of the presented calculation method for *m*_0_, spoiler not opened, *t* = 0 s, *β* = 0°, *f*_r_ = 10 GHz, HH, drawn using MATLAB 2015A.

Figure 8 reveals that the simultaneous deflection of the spoiler on both sides has a greater impact on the electromagnetic scattering characteristics of the aircraft than the deflection of the single-sided spoiler. When *β* = 10°, the main differences of the three RCS curves are widely reflected in the azimuth range of 18.5°–40.25°, 67°–104.8°, and 231.5°–292.3°, where the mean value of the RCS curve when *A*_s1_ = *A*_s2_ = 30° is −5.7055 dBm^2^, and that at *A*_s1_ = *A*_s2_ = 50° is −5.2140 dBm^2^, as shown in Table 3. Refer to Figure A2 and Figure A3 in Appendix A for more detailed RCS data. For *β* = 10°, the RCS level of the *A*_s1_ = *A*_s2_ = 90° curve is significantly higher than the other two, where the mean of the RCS curve at *A*_s1_= *A*_s2_ = 30° is −6.2461 dBm^2^, and those at *A*_s1_ = *A*_s2_ = 60° and *A*_s1_ = *A*_s2_ = 90° are −5.9529 dBm^2^ and −5.5647 dBm^2^, respectively. The main reason for this increase in RCS is the presence of two vertical spoilers on the surface of the aircraft, which allows more strong scattering sources in a large range of azimuth angles in the head and side directions. These results show that when the two spoilers act at the same time, the effect on the aircraft RCS curve and the mean value is obvious.

**Figure 9:**
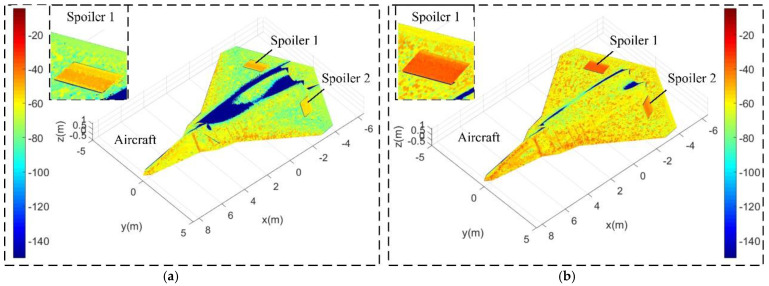
Surface scattering characteristics of the aircraft, *K*_cd_ = 1, HH, M0 mode, color window [−150, −5], RCS unit: dBm^2^, drawn using MATLAB 2015A. (**a**) *α* = 10°, *β* = 5°, *A*_s1_ = *A*_s2_ =1 9.8°. (**b**) *α* = 19.75°, *β* = 15°, *A*_s1_ = *A*_s2_ = 46.8°.

Figure 9 provides that the surface scattering characteristics of the spoiler at different deflection angles are obviously different from those of other parts of the aircraft under certain important observation angles. When the spoiler deflects 19.8°, the current incident wave produces brightly colored scattering areas on the surface of the aircraft, where a little orange (about −50 dBm^2^) appears on the leading edge of the wing, the edge of the nose, and part of the cockpit surface, while the scattering intensity of the upper surface of the two spoilers is significantly higher than the nearby wing surface, because the angle between the wing and the incident wave is very small at this time, the deflection effect on the radar wave is very weak, and the spoiler that opens upward can be well illuminated by electromagnetic waves. When *α* = 19.75°, *β* =1 5°, and *A*_s1_ = *A*_s2_ = 46.8°, most areas of the aircraft surface are illuminated by incident waves, where the orange on the top of the cockpit, the edge of the front fuselage, and the upper surface of the spoiler has been deepened, and some are even deepened to red (increases to about −30 dBm^2^). Note that the color of the upper surface of the spoiler is relatively concentrated, where one is orange red (about −35 dBm^2^) and the other is light orange (about −55 dBm^2^). This is because the entire surface comes from the rear part of the wing with almost the same curvature, which, in turn, has a similar effect on the deflection of the radar wave. These results indicate that the deflected spoiler may make it a strong scattering source for aircraft under certain observation angles.

### 4.3. Linear Dynamic Deflection

**Figure 10:**
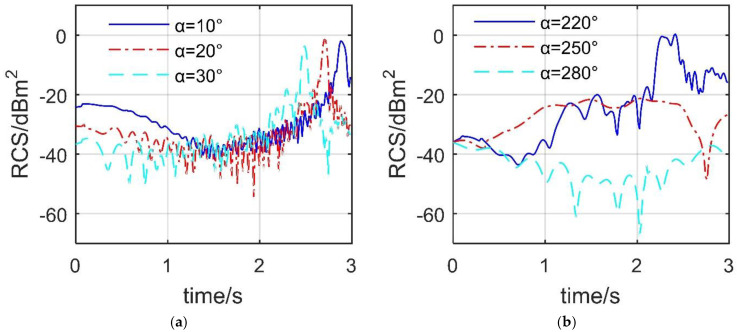
Dynamic RCS of spoiler 1 when it deflects independently, M1 mode, HH, *ω*_s1_ = 0.5236 rad/s, drawn using MATLAB 2015A. (**a**) RCS at *β* = 0°, (**b**) RCS at *β* = 5°.

Figure 10 presents that the dynamic RCS of spoiler 1 under different azimuth angles is quite different. When *β* = 0°, the maximum fluctuation amplitudes of the three RCS curves are similar, while the peaks appear gradually in advance as the azimuth angle increases from 10° to 30°. The peak value of the RCS curve at *α* = 10° is −1.907 dBm^2^, which appears at 2.88 s, at this time, the spoiler deflects 86.4°, while the RCS peak at *α* = 30° is −3.715 dBm^2^, appearing at 2.49 s. As the azimuth angle increases from 10° to 30°, the angle between the incident wave and the deflection axis of the spoiler 1 gradually tends to be a right angle, which makes the upper surface of the spoiler easier to form a large-area strong scattering source. For *β* = 0°, the dynamic RCS curves under the three azimuth angles are very different, including shape, fluctuation amplitude, and peak value. The average value of the RCS curve under *α* = 220° is −11.5657 dBm^2^, while that under *α* = 250° and 280° is −25.1756 dBm^2^ and −41.6775 dBm^2^, respectively. This is because, compared with the other two cases, the angle between the 220-degree azimuth and the normal plane of the spoiler shaft is smaller, which makes the lower surface of the spoiler less effective in deflecting radar waves. These results show that the RCS dynamic response of the spoiler’s deflection to itself is significant.

**Figure 11:**
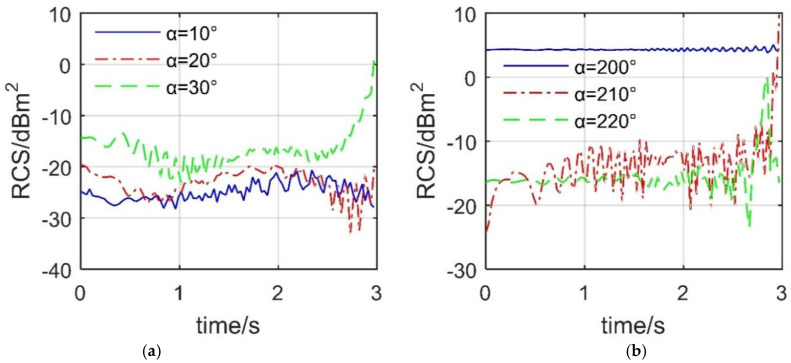
Dynamic RCS of the aircraft, ω_s1_ = ω_s2_ = 0.5236 rad/s, M1 mode, HH, drawn using MATLAB 2015A. (**a**) RCS at *β* = 0, (**b**) RCS at *β* = 5°.

Figure 11 shows that when the two spoilers are deflected at the same time, it will also have a significant impact on the dynamic RCS of the aircraft at certain azimuth angles. For the case of *β* = 0°, the fluctuation level of the RCS-*t* curve under *α* = 10° and 20° is lower than that of the RCS-*t* curve under *α* = 30°, where the average value of the RCS curve with *α* = 30° reaches −13.7925 dBm^2^. At *t* = 2.94 s, the RCS of *α* = 30° curve increases to −5.628 dBm^2^, which is an increase of 8.702 dBm^2^ compared to the initial value. This is a considerable increase, because the presence of two spoilers increases the scattering source of the aircraft surface, and the deflection angle reaches 88.2°, which is not conducive to deflecting the incident wave to a non-threatening direction.

For the case of *β* = 5°, it can be seen that the RCS curve under *α* = 200° fluctuates slightly around 4.289 dBm^2^. The maximum RCS difference of the curve under *α* = 210° is 34.1761 dBm^2^, and that under *α* = 220° is 23.7239 dBm^2^, where the former has a mean value of −7.9392 dBm^2^, and the latter has a mean value of −13.3698 dBm^2^. At *t* = 2.85 s, the RCS curve under *α* = 220° reaches the maximum value of 0.214 dBm^2^, which is an increase of 16.694 dBm^2^ compared to the initial value, because at this time, the lower surface of spoiler 1 and the inner side of spoiler 2 make major contributions to this RCS increase. These results indicate that the deflection of the spoiler can destroy the original stealth characteristics of the aircraft at certain azimuth angles.

**Figure 12:**
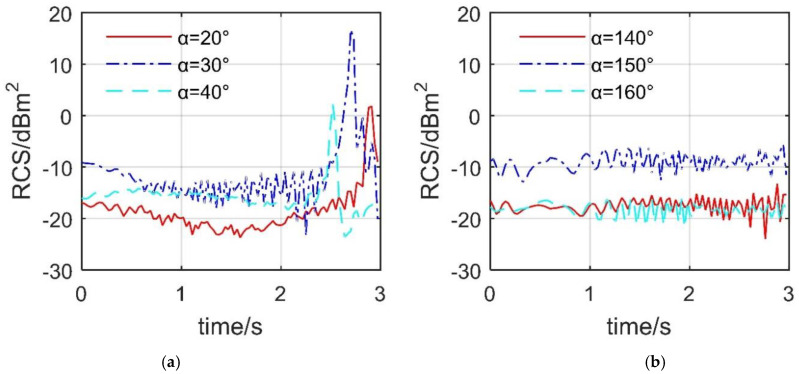
Dynamic RCS response of the aircraft, HH, *ω*_s1_ = *ω*_s2_ = 0.5236 rad/s, M1 mode, drawn using MATLAB 2015A. (**a**) RCS at *β* = 10°, (**b**) RCS at *β* = 15°.

Figure 12 indicates that the deflection of the spoiler will still have a dynamic impact on the aircraft’s RCS at the given observation angle. For the case of *β* = 10°, the three RCS curves all show huge fluctuations, and the maximum difference of their respective RCS exceeds 25.844 dBm^2^. The peak values of these three RCS curves are also different, where the peak value of the RCS curve under *α* = 30° is 16.69 dBm^2^ appearing at *t* = 2.7 s. Although the opening process of the spoiler is only a few seconds, this action will have a very unfavorable dynamic impact on the RCS of the aircraft under certain important azimuth angles. This short and violent change is also fatal for advanced tailless fighters. For the case of *β* = 15°, the three RCS curves all show more small fluctuations with an amplitude of around 6.72 dBm^2^. The level of the RCS curve under *α* = 140° and *α* = 160° is similar, but lower than that of *α* = 150°. For the RCS curve at *α* = 150°, it only takes 1.14 s to increase the aircraft’s RCS from −8.875 dBm^2^ to −6.491 dBm^2^. This is also a considerable increase, because at this time, the lower surface of spoiler 2 has not been fully opened up, which has already brought a large RCS change to the aircraft. These results show that, under the current elevation angle, the dynamic RCS response caused by the spoiler deflection of the aircraft at important azimuth angles is also obvious.

### 4.4. Smooth Dynamic Deflection

**Figure 13:**
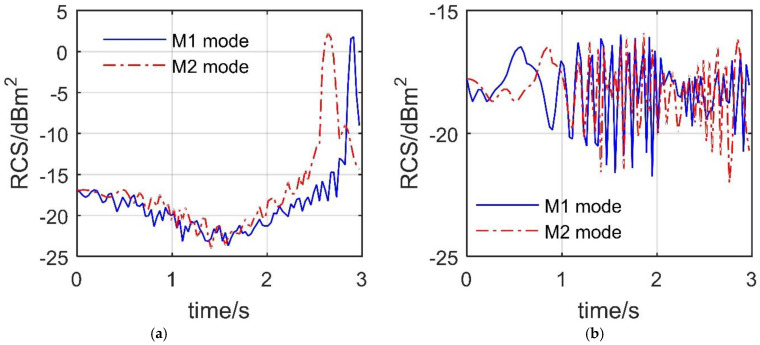
Dynamic RCS response of the aircraft, *β* = 10°, *A*_k_ = *C*_k_ = π/4, *ω*_k_ = 1.0472 rad/s, M2 mode, drawn using MATLAB 2015A. (**a**) RCS at *α* = 20°, *β* = 10°, (**b**) RCS at *α* = 160°, *β* = 50°.

Figure 13 reveals that the influence of different dynamic deflection modes on the RCS curve at the given observation angle is mainly reflected in the peak position and local fluctuations. For the RCS at *α* = 20° and *β* = 10°, the mean value of the curve in M1 mode is −13.0187 dBm^2^, and that of the curve in M2 mode is −10.8438 dBm^2^. This indicates that the M2 mode could significantly reduce the average level of RCS under the current observation angle, which is beneficial to the stealth of the aircraft. The peak of the curve with M1 mode is 1.799 dBm^2^ at 2.91 s, and that of the curve with M2 mode is 2.216 dBm^2^ at 2.64 s. The peak position is advanced, because the increase rate of the deflection angle in M2 mode first increases and then decreases, where the spoiler has been deflected by 86.8399° when *t* = 2.64 s. For the RCS at *α* = 160° and *β* = 50°, the fluctuation ranges of the two curves are similar, where the RCS mean at M1 mode is −18.0246 dBm^2^, and that at M2 mode is −18.0868 dBm^2^. This shows that, under the current azimuth angle (tail direction, *α* = 160°), the difference in the influence of the two deflection modes on the aircraft RCS is mainly reflected in the local fluctuations. It can be seen that the use of M2 mode is beneficial for the aircraft to stealth in the forward direction (*α* = 20°).

**Figure 14:**
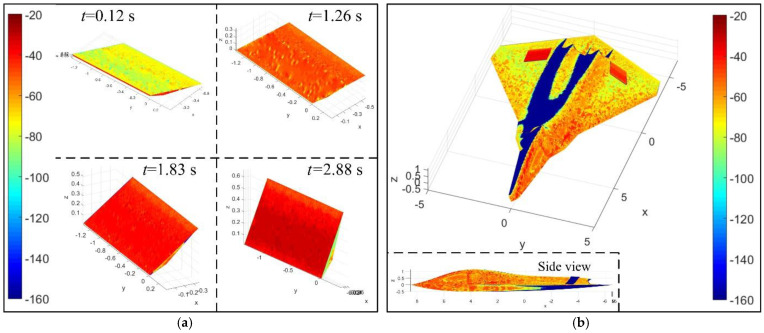
Surface scattering characteristics, M2 mode, *K*_cd_ = 1, RCS unit: dBm^2^, *A*_k_ = *C*_k_ = π/4, *ω*_k_ = 1.0472 rad/s, M2 mode, color window [−160, −20], drawn using MATLAB 2015A.

Figure 14 investigates that, under M2 mode, the scattering intensity of the spoiler surface is lower in the first half of the time and higher in the second half of the time. When *t* = 0.12 s, this spoiler is deflected by only 0.3548°, where the upper surface of the spoiler shows a staggered distribution of green (about −90 dBm^2^) and yellow (about −70 dBm^2^). Note that the trend toward red indicates that the scattering intensity here is high, and the trend toward blue indicates that the scattering level here is low. At this time, the narrow edges and sides below the leading edge are lit by the incident wave, showing a distinct red (about −30 dBm^2^). At *t* = 1.26 s, the deflection angle of the spoiler quickly increased to 33.809°, where the yellow on the top surface of the spoiler has almost disappeared, replaced by a large area of light red (about −50 dBm^2^), that is, the scattering level is reduced by 20 dBm^2^. At this time, the side of the spoiler turns into red and yellow inclusions. When *t* = 1.83 s, the spoiler is deflected by 60.2432°, where the upper surface of the spoiler is almost entirely red, with a small amount of blue on the side. At *t* = 2.88 s, the deflection angle of the spoiler is 89.6452°, where the side edge of the spoiler appears green, while the upper surface of the spoiler is dark red and red. For the aircraft, the spoiler is deflected by 87.7975° when the time is equal to 2.7 s. The upper surface of spoiler 1 is darker in red than that of spoiler 2, where the scattering level of these two spoilers is significantly higher than that of the nearby curved surface, because at the current azimuth, the angle between the incident wave and the upper surface of spoiler 1 is greater than the angle between the incident wave and the upper surface of spoiler 2. This makes spoiler 1 unfavorable to deflect the current incident wave to a non-threatening direction. Other high-scattering areas include the side-illuminated part of the fuselage head, the top of the cockpit, and the curved surface near the edge of the front fuselage. These results indicate that the scattering level of the spoiler in the second half of the M2 mode is undesirable.

## 5. Conclusions

The presented method is established to evaluate the electromagnetic scattering characteristics of the aircraft. By studying the influence of spoiler deflection on the stealth characteristics of the advanced tailless fighters, the following conclusions can be made:

(1) According to the current design of the spoiler, the spoiler opened at a large angle will cause strong scattering sources under the head and tail incident waves. As the elevation angle increases, the influence of the spoiler on the side electromagnetic scattering characteristics of the aircraft becomes more obvious;
(2) The influence of spoiler deflection on its dynamic RCS is extensive and obvious, including the key azimuth angles of head, side, and tail. When the spoilers on both sides deflect, it brings very unfavorable dynamic changes to the RCS under the key azimuth angles of the head and tail of the aircraft;
(3) Compared with the M1 mode, the M2 mode is conducive to the reduction in the average RCS at the given head azimuth, while the scattering level on the upper surface of the spoiler is high in the second half of the time.

## Figures and Tables

**Table 1:** The size data distribution of each part of the aircraft.

*L*_airc_ (m)	*W*_airc_ (m)	*H*_airc_ (m)	*A*_fe_ (°)	*A*_wt_ (°)	*A*_le_ (°)	*L*_af_ (m)	*L*_f1_ (m)	*L*_f2_ (m)	*L*_f3_ (m)	*W*_sc_ (m)	*W*_se1_ (m)
15	10	1.6646	72.475	8.68	27.924	8.5	4.2112	6.6106	11.24	0.751	3.1

**Table 2:** The grid size distribution of each part of the aircraft.

Area	Max Size (mm)	Area	Max Size (mm)
Global minimum size	2	Spoiler leading edge	3
Spoiler trailing edge	5	Edge of aircraft nose	6
Wing leading edge	10	Trailing edge of wing	8
Inlet edge	10	Inlet face	15
Nozzle face	8	Spoiler cabin edge	30
Wing	50	Exhaust port	35
Fuselage	75	Aircraft	100

**Table 3:** RCS mean of the aircraft with fixed deflection of bilateral spoiler, HH.

	*Β* = 10°			*Β* = 15°		
*A*_s_ (°)	20	30	50	30	60	90
Mean (dBm^2^)	−6.0904	−5.7055	−5.2140	−6.2461	−5.9529	−5.5647

## Data Availability

Not applicable.

## Appendix A

For spoiler 2: noting the symmetry of the two spoilers with respect to the *x–z* plane, the translation operation can be written as:

(A1) $$M^{x}\left(m_{s2}\left(t=0\right)\right)=M\left(x\left(m_{s2}\left(t=0\right)\right)-x\left(P_{s2}\right)\right)$$

(A2) $$M^{xy}\left(m_{s2}\left(t=0\right)\right)=M^{x}\left(y\left(m_{s2}\left(t=0\right)\right)-y\left(P_{s2}\right)\right)$$

(A3) $$M^{xyz}\left(m_{s2}\left(t=0\right)\right)=M^{yz}\left(z\left(m_{s2}\left(t=0\right)\right)-z\left(P_{s2}\right)\right)$$

where *m*_s2_ is the model of spoiler 2, and *P*_s2_ is the symmetric point of *P*_s1_ about the *x–z* plane. Rotate the obtained spoiler 2 model around the coordinate axis:

(A4) $$M^{r,z}\left(m_{s2}\left(t=0\right)\right)=\left[\begin{array}{ccc} \cos\left(-A_{z1}\right) & -\sin\left(-A_{z1}\right) & 0\\ \sin\left(-A_{z1}\right) & \cos\left(-A_{z1}\right) & 0\\ 0 & 0 & 1 \end{array}\right]\cdot M^{xyz}\left(m_{s2}\left(t=0\right)\right)$$

(A5) $$M^{r,x}\left(m_{s2}\left(t=0\right)\right)=\left[\begin{array}{ccc} 1 & 0 & 0\\ 0 & \cos\left(-A_{x1}\right) & -\sin\left(-A_{x1}\right)\\ 0 & \sin\left(-A_{x1}\right) & \cos\left(-A_{x1}\right) \end{array}\right]\cdot M^{r,z}\left(m_{s2}\left(t=0\right)\right)$$

When the spoiler 2 deflects upward, its dynamic model can be expressed as:

(A6) $$M^{r,y}\left(m_{s2}\left(t\right)\right)=\left[\begin{array}{ccc} \cos\left(-A_{s2}\left(t\right)\right) & 0 & -\sin\left(-A_{s2}\left(t\right)\right)\\ 0 & 1 & 0\\ \sin\left(-A_{s2}\left(t\right)\right) & 0 & \cos\left(-A_{s2}\left(t\right)\right) \end{array}\right]\cdot M^{r,x}\left(m_{s2}\left(t=0\right)\right)$$

It could be seen that the overall trend and peak value of the two RCS curves are consistent, as shown in Figure A4. The RCS difference between the azimuth angles of 39.75°–61° and 286°–298.5° is obvious, while the average value of the RCS curve determined by PO+MOM/MLFMM is 7.3302 dBm^2^, which is 0.2047 dBm^2^ larger than the other one. This figure represents the verification of RCS azimuth calculation results by the presented calculation method at the given moment, noting that the RCS results in this paper mainly include RCS azimuth and RCS time. These results show that the presented calculation method is feasible and accurate when used to evaluate the electromagnetic scattering characteristics of the aircraft.
